# A Novel Porous PDMS-AgNWs-PDMS (PAP)-Sponge-Based Capacitive Pressure Sensor

**DOI:** 10.3390/polym14081495

**Published:** 2022-04-07

**Authors:** Xueqiang Tan, Jimin Zheng

**Affiliations:** College of Chemistry, Beijing Normal University (BNU), Beijing 100875, China; tan_xueqiang@126.com

**Keywords:** porous PAP sponge, capacitive pressure sensor, sensitivity, PDMS, AgNWs

## Abstract

The development of capacitive pressure sensors with low cost, high sensitivity and facile fabrication techniques is desirable for flexible electronics and wearable devices. In this project, a highly sensitive and flexible capacitive pressure sensor was fabricated by sandwiching a porous PAP sponge dielectric layer between two copper electrodes. The porous PAP sponge dielectric layer was fabricated by introducing highly conductive silver nanowires (AgNWs) into the PDMS sponge with 100% sucrose as a template and with a layer of polydimethylsiloxane (PDMS) film coating the surface. The sensitivity of the PAP sponge capacitive pressure sensor was optimized by increasing the load amount of AgNWs. Experimental results demonstrated that when the load amount of AgNWs increased to 150 mg in the PAP sponge, the sensitivity of the sensor was the highest in the low-pressure range of 0–1 kPa, reaching 0.62 kPa^−1^. At this point, the tensile strength and elongation of sponge were 1.425 MPa and 156.38%, respectively. In addition, the specific surface area of PAP sponge reached 2.0 cm^2^/g in the range of 0–10 nm pore size, and showed excellent waterproof performance with high elasticity, low hysteresis, light weight, and low density. Furthermore, as an application demonstration, ~110 LED lights were shown to light up when pressed onto the optimized sensor. Hence, this novel porous PAP-sponge-based capacitive pressure sensor has a wide range of potential applications in the field of wearable electronics.

## 1. Introduction

The development of flexible electronics applications in recent decades has created an explosion in demand for cost-effective sensors capable of detecting an array of inputs including strain, pressure, temperature, humidity, and the presence of chemical compounds. Flexible electronic devices are typically built onto a flexible polymer substrate, or elastomer. Polydimethylsiloxane (PDMS) is one of the most commonly used elastomer materials and is widely employed in functional coatings [1,2,3,4,5,6], wearable devices [7,8,9,10,11], solar cells [12,13,14,15,16], medical systems [17,18,19], biomimetic materials [20,21,22,23,24,25,26,27,28,29], flexible electronic devices [30,31,32,33,34,35,36,37], microfluidic chips [38,39,40,41], robotics [42,43,44], etc. Compared with other types of elastic substrates such as hydrogels and polyurethanes, PDMS has unique advantages, such as its applicable temperature range (−100 °C to 200 °C) and chemical stability (acid, alkali and oil resistance), which gives PDMS elastomers excellent reliability and durability. However, PDMS has relatively poor inherent mechanical properties (modulus, strength), which limits its bearing capacity under external load. In order to compensate for these defects, researchers usually emphasize designing new physics structure or adding fillers to improve the mechanical and thermal properties, electrical properties, self-healing functions and wettability of PDMS.

Recently, porous PDMS (pPDMS) sponges have been applied in oil spill cleaning [45,46,47,48], cell culture [49,50,51,52], photocatalysis [1,53,54], flexible sensors [55,56,57,58], triboelectric nanogenerators and other fields [59,60,61] due to their relatively high specific surface area, low density, low surface energy, excellent hydrophobicity and compressibility. Song et al. [62] have recently developed a compressible carbon nanotube–polydimethylsiloxane (CNT-PDMS) sponge with high piezoresistive sensitivity and stable electrochemical performance and a simple, low-cost production method. CNT-PDMS also has considerable mechanical advantages as it maintains stable electrochemical performance even under high compressive strain (50%) and can be directly attached to the skin or clothing, such as in a wearable health monitoring device. Wang et al. [63] used a sugar template to create a bi-continuous porous structure in the sponge. Such a porous morphology and the inherent hydrophobicity of PDMS provided the sponge with up to 19.90 times oil absorption capacity. In addition, this study illustrated that the adsorption capacity and separation performance of the PDMS sponge was closely related to the porosity of the material which could be controlled by adjusting the sugar load. Importantly, the addition of 2 wt.% poly (dimethylsiloxane-b-ethylene oxide) (PDMS-b-PEO) to the system could significantly change the surface properties of the sponge, thus affecting the wettability and separation behavior of the sponge. The modified sponge was used for circulating filtration, and the oil removal rate reached 99.9%, which indicated that the modified sponge had stable and efficient separation performance.

Pan et al. [64] developed a conductive graphene-embedded PDMS sponge using porous sugar cubes treated with small amounts of graphene. This graphene-PDMS sponge showed improved mechanical tolerance, achieving 80% strain with only 0.4 MPa, which was half of the stress required for a PDMS sponge. However, graphene has limited conductivity compared to silver nanowires, which not only benefit from the excellent conductivity of silver, but also have excellent light transmittance, bending resistance and flexibility. It is worth noting that research on PAP sponges with AgNWs as fillers remains limited. Therefore, a AgNWs-embedded capacitive sensing material with a simple fabrication process, excellent repeatability and high sensitivity would be a significant development in the field.

In this research, a novel porous PDMS-AgNWs-PDMS (PAP)-sponge-based capacitive pressure sensor was fabricated successfully. The pressure sensor consisted of a soft porous PAP sponge dielectric layer and two copper conductive electrodes. One-dimensional AgNWs, as excellent conductive fillers, were loaded on the sponge. The PDMS sponge was prepared using sucrose as a template and a layer of PDMS film was coated on the surface to obtain PAP sponge. In order to further improve the sensitivity of the sensor, this project employed a simple and environmentally friendly method to increase the load amount of AgNWs in the PAP sponge. The experimental results showed that the sensitivity of PAP sponge capacitive pressure sensor was as high as 0.62 kPa^−1^ in the low-pressure range. In addition, PAP sponge was also shown to have the advantages of high elasticity, low hysteresis, light weight, excellent mechanical properties, low density and good waterproof performance. The optimized PAP sensor was able to light up over 110 LED bulbs, demonstrating considerable potential in the application of flexible and wearable devices.

## 2. Materials and Methods

### 2.1. Materials

Sucrose and soft white sugar (analytical pure), Beijing Chemical Works (Beijing, China); PDMS, Dow Corning DC184 (Sylgard 184, Dow Corning); AgNWs ethanol dispersion (concentration: 20 mg/mL, diameter: 50 nm, length: 5 µm), Guangzhou Nano Chemical Technology Co., Ltd. (Guangzhou, China).

### 2.2. Preparation of Porous PDMS Sponge

In this project, the template of the PDMS sponge was prepared with sugar since it has excellent chemical stability, and the pore size of the sponge can be adjusted using various sizes of sugar. Quantities of 20 g of sugar and 5 g PDMS (4.5 g PDMS + 0.5 g curing agent) were combined in a 50 mL beaker and stirred. PDMS and sugar were stirred thoroughly to ensure even mixing. The mixture was then cured in an oven at 60 °C for 4 h. After curing, the beaker was filled with deionized water and the sugar template was allowed to dissolve at room temperature for 24 h, followed by ultrasound (3 kW) treatment with a frequency of 40 kHz for 2 h. Finally, the template was removed from the ultrasound bath, and the soft PDMS sponge was dried in an oven at 60° C for 12 h. The sugar templates included soft white sugar and sucrose. This process was repeated with 0%, 33%, 50%, 67% and 100% soft white sugar content in the 20 g sugar component of the template.

### 2.3. Preparation of PAP Sponges

AgNWs ethanol dispersion (25 mL) was dropped onto the dry sponge through a dropper and the sponge was pressed repeatedly until the AgNWs were evenly dispersed in the sponge. The sponge was then dried in an oven at 60 °C for 12 h. The resultant AgNWs-PDMS sponge was immersed in PDMS solution for 12 h to coat it with a thin layer of PDMS film, and finally PAP sponges were prepared. Among them, four samples with 100% sucrose as template and AgNWs loading capacities of 50 mg (P-1), 100 mg (P-2), 150 mg (P-3) and 200 mg (P-4) were produced.

### 2.4. Characterization of the PAP Sponge

The energy spectrum mapping and morphology of samples were tested using a Zeiss G300 scanning electron microscope (Dongguan Sanben Precision Instrument, Dongguan, China). The specific surface and porosity of PAP sponge were measured using a Mack 2020 HD88 (BET) (Shanghai McMurraytick Instruments, Shanghai, China). The infrared spectrum of samples was obtained via a high temperature synchronous thermal analyzer (RT-1600) (STA) (Beijing Hengjiu Experimental Equipment, Beijing, China).

In this project, the mechanical properties of PAP sponge during stretching were tested by universal material testing machine (Japan Shimadzu AGS-X5KN, Kyoto, Japan). A length of 0.5 cm of the PAP sponge sample was clamped at both ends on the collet of the universal material testing machine. Because the elastic modulus of the PAP sponge was much smaller than that of the metal collet, overly tight clamping would lead to the fracture of the PAP sponge sample while too loose clamping would lead to the PAP sponge sample falling off the collet during stretching, so it was necessary to add a piece of rubber pad on both sides of the collet. The universal material testing machine recorded the external force applied in the whole process from the beginning of stretching of the sample to breaking, with further analysis undertaken to obtain the tensile strength and elongation of PAP sponge. Real-time output capacitance of the PAP sponge capacitive pressure sensor was measured using an impedance analyzer (WK6500B) (Shanghai Ruixuan Electronic Technology, Shanghai, China). Wires attached at both ends of the pressure sensor were connected to the impedance analyzer. To maintain the same external force and frequency during capacitive pressure sensor implementation, a controllable tapping system, combining an air compressor (Beijing Bomairui Compression Machinery, Beijing, China) and an intelligent pneumatic punch (Taizhou Ruite Machinery Equipment, Taizhou, China), was used to drive the PAP-sponge-based capacitive pressure sensor operation.

## 3. Results and Discussion

**Figure 1:**
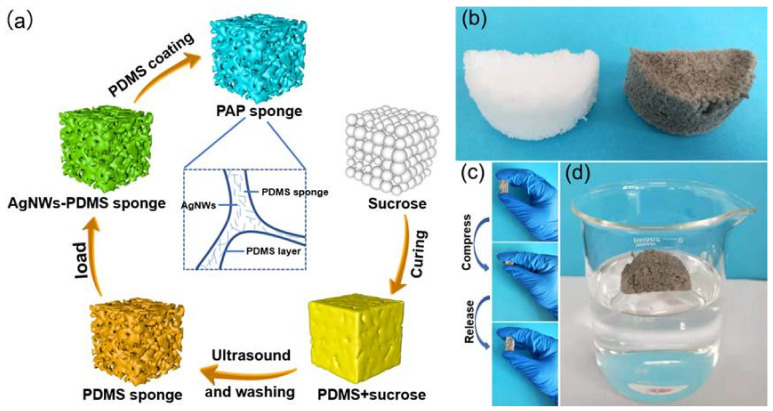
(**a**) Schematic illustration of the fabrication processes of PAP sponge. Digital photographs of (**b**) the PDMS sponge and PAP sponge, (**c**) the PAP sponge under a compressing and releasing cycle, and (**d**) the PAP sponge floating on the water surface.

As summarized in Figure 1a, a PAP sponge was prepared using sucrose to provide a template for the PDMS sponge. After curing the PDMS-sugar mixture, the sugar was removed via deionized water and ultrasound. The resultant soft porous PDMS sponge was then dried and saturated with an ethanol dispersion of AgNWs. The AgNWs-PDMS sponge was then immersed in PDMS solution to coat the sponge in a thin layer of PDMS film. After further curing, the PDMS coating acted as a binder enhancing the interfacial adhesion between the AgNWs and the PDMS sponge skeleton. If there had been no PDMS coating, AgNWs would have fallen off the surface of the material due to weak interfacial interaction.

As shown in Figure 1b, following removal of the sugar template, the PDMS sponge was white, and turned grey once the AgNWs was loaded. As shown in Figure 1c, the loaded PAP completely recovered from compression deformation. This indicated that the introduction of AgNWs did not reduce the flexibility and compressibility of the sponge. Figure 1d illustrates that the PAP sponge composite exhibited waterproof performance with high elasticity, light weight, low density (0.486 g/cm^3^), and could float at the water-air interface.

**Figure 2:**
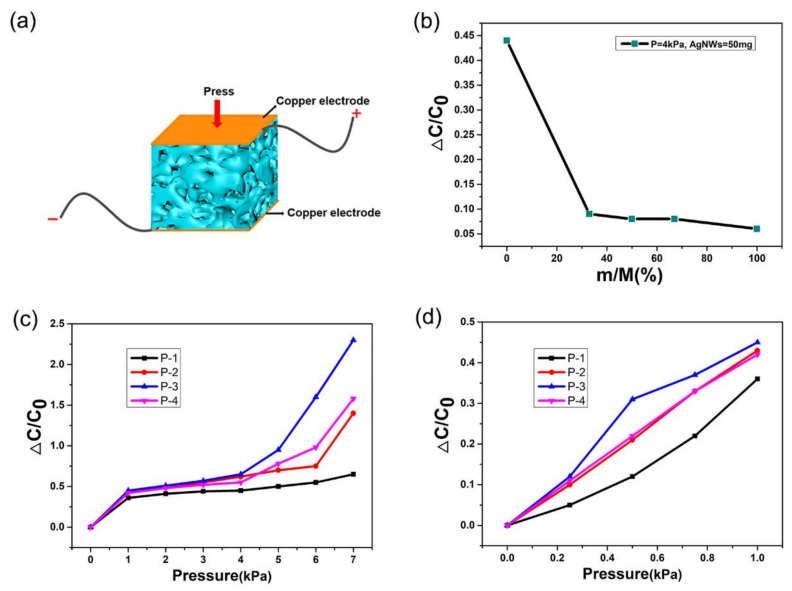
(**a**) Schematic of the PAP sponge capacitive pressure sensor structure. (**b**) The change curve of the capacitance change rate of PAP sponge by content of soft white sugar in the template. (**c**,**d**) Sensitivity curves of the capacitive pressure sensor with different load amounts of AgNWs under applied pressure.

First, to optimize the doping ratio of soft white sugar, the response performance of the capacitive pressure sensors was characterized. In this study, sucrose and soft white sugar were used as templates to prepare samples with 0%, 33%, 50%, 67% and 100% soft white sugar content. The configuration of the PAP sponge capacitive pressure sensor is depicted in Figure 2a. Copper electrodes were pasted on both ends of the PAP sponge and led out the positive and negative wires. The sensitivity S of the pressure sensor can be defined as:

(1) $$S=\frac{\left(\frac{\left(C-C_{0}\right)}{C_{0}}\right)}{\Delta P}$$

where Δ*P* is the applied pressure, *C*_0_ and *C* refer to the initial capacitance and the capacitance under pressure, respectively (Equation (1)). Applying a pressure of 4 kPa to the PAP sponge, quantities of 50 mg AgNWs were loaded into sponges with various mixing ratios of soft white sugar. Capacitance was measured and the capacitance change rate, Δ*C/C*_0_, was calculated according to Equation (2):

(2) $$\frac{\Delta C}{C_{0}}=\frac{C-C_{0}}{C_{0}}\;$$

The calculated results were used to generate curves of the capacitance change rate of PAP sponges with 50 mg AgNWs capacity at different soft white sugar concentrations as shown in Figure 2b. The soft white sugar content, *m/M*, was defined as the amount of soft white sugar, *m*, over the total mass of the sugar template, *M*. Higher soft white sugar content resulted in a lower capacitance change rate and the capacitance change rate peaked at 0.45 when the template was purely sucrose. The experimental results showed that the sensor with a 100% ratio of sucrose template exhibited the highest sensitivity. When the pressure was 4 kPa, the sensitivity of the pure sucrose template (0.1125 kPa^−1^) was about six times higher than that of the pure soft white sugar template (0.019 kPa^−1^). Therefore, a sucrose doping ratio of 100% was chosen as the optimal template for the PAP sponge capacitive pressure sensor.

To further improve the sensitivity of the capacitive pressure sensor, PAP sponges with increased load amounts of AgNWs were produced. PAP sponges made with 100% sucrose templates were prepared as previously described and loaded with 50 mg (P-1), 100 mg (P-2), 150 mg (P-3) and 200 mg (P-4) AgNWs. Then, relevant characterization of the four samples was performed. Figure 2c,d shows the sensitivity curves of the capacitive pressure sensor for different AgNWs loads. It can be clearly seen that the P-3 sensor with a load amount of 150 mg AgNWs exhibited the highest sensitivity—as high as 0.62 kPa^−1^ in the 0–1 kPa low-pressure range. Although the sensitivity decreased at higher pressures, it still reached 0.45 kPa^−1^ in the pressure range of 1–7 kPa, which was comparable to that reported for many capacitive pressure sensors [65,66]. Similarly, the sensitivity of the P-1 sensor with a load of 50 mg AgNWs was 0.36 kPa^−1^ in the 0–1 kPa low-pressure range and decreased to 0.092 kPa^−1^ with increased pressure. Compared with the other types of pressure sensors shown in Table 1, the as-prepared capacitive pressure sensors had better performance, such as higher sensitivity [65,66,67,68,69,70]. However, sensitivity was lower in the PAP sponges with 200 mg AgNWs loaded, indicating that this effect did not scale linearly. Piezoresistive sensors have the disadvantages of poor stability and a large hysteresis effect, while piezoelectrical sensors have the disadvantage of low spatial resolution. Assuming the above shortcomings are overcome, capacitive pressure sensors have many unique advantages [65,66,67,68,69,70], including the following: (1) Capacitive pressure sensors show high accuracy in detecting static loads; (2) They possess characteristics of high sensitivity, low power consumption, low hysteresis effects and a wide response range; (3) Compared with the other two sensors, the capacitive sensor was shown to be easy to operate and simplified the design and analysis process of the device. The excellent sensitivity of the sensor at low-pressure range, combined with a wide sensing range, makes it possible to detect slight touch information. These results indicate that the sensing performance of the fabricated capacitive pressure sensor based on PAP sponge structure was successfully enhanced by optimizing the loading amount of AgNWs.

AgNWs have the advantages of high mechanical strength, high dielectric coefficient, excellent conductivity, and large specific surface area. They have been widely used in flexible electronic devices. As the core part of the sensor, AgNWs have the following three main functions in composites: the first function is to turn on the circuit. When an external force is applied to the sensor, AgNWs are interconnected to form a conductive path. When the distance between AgNWs is too large and electrons cannot produce a quantum tunneling effect, the PDMS sponge between adjacent AgNWs will form the structure of the capacitor. Currently, AgNWs-PDMS composites show the characteristics of equivalent capacitance. The second function is to change the relative dielectric constant *ε_r_* to improve the sensitivity and response range of the sensor. For further detailed analysis and demonstration, please refer to the experiment and discussion below.

**Figure 3:**
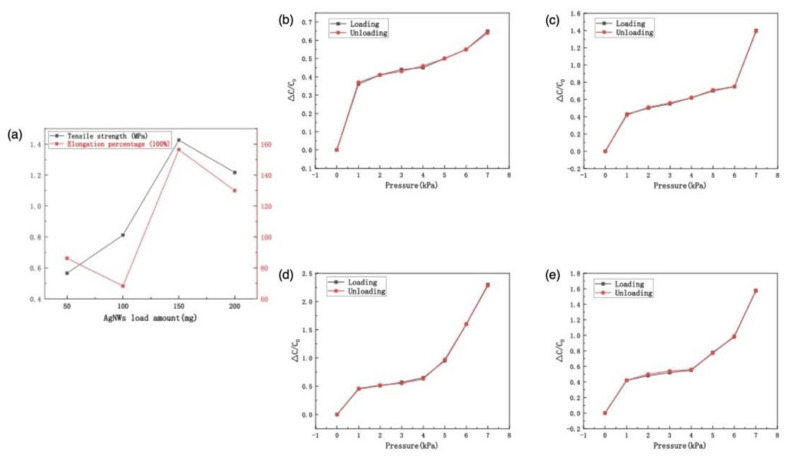
(**a**) The tensile strength and elongation percentage curve of four samples; The loading pressure curve and unloading pressure curve of the four sensors (**b**) P-1; (**c**) P-2; (**d**) P-3; (**e**) P-4.

In addition to the above two functions, more importantly, AgNWs loaded in PDMS sponge can improve the mechanical properties of PAP sponge [71]. As shown in Figure 3a, the maximum tensile strength of AgNWs-PDMS sponge first increased and then decreased with increase in AgNWs load, from 0.565 MPa at 50 mg to 1.425 MPa at 150 mg, and then decreased to 1.216 MPa at 200 mg. The elongation of sponge fluctuated with the increase in AgNWs load and reached a peak when the load was 150 mg, which was 156.38%. The loading amount of AgNWs in the P-4 sample was the largest (200 mg) but its elongation was lower than that of the P-3 sample (150 mg). The main reason was that in the P-4 sample, more interconnected AgNWs led to stress concentration, so it was more easily pulled apart [72]. This indicated that the P-3 sensor was more suitable for detecting scenes with large strain.

In order to test whether the sensor has hysteresis, the loading curve and unloading curve of the four sensors were tested under the same pressure change. As shown in Figure 3b–e, the response curves of the four sensors, when applying and removing the pressure, were basically consistent and there was no obvious height difference between the loading curve and the unloading curve. In addition, in the process of loading and unloading pressure, the detection signal of the sensor was consistent. The above experimental results show that the sensors assembled with four samples had no obvious hysteresis effects, the dielectric layer could be effectively restored to its original state when the pressure was removed, and that there was less viscosity in the structure.

Subsequent experiments were performed using the P-3 sponge as the optimal dielectric layer of the flexible capacitive pressure sensor which was shown to have the best sensitivity range of the sponges produced. When external pressure acted on the sensor, the distance between the upper and lower electrodes of the sensor changed, as did the distance between the silver nanowires adsorbed inside the sponge. The change of dielectric constant of the AgNWs/PDMS composite sponge under pressure can be interpreted according to the Kirkpatrick and Zallen statistical percolation model [73,74] which is used to predict the electrical properties of a percolation system with non-interacting randomly dispersed fillers. Here, the capacitance, *C*, of a parallel plate capacitator is defined in Equation (3) as:

(3) $$C=\varepsilon_{0}\varepsilon_{r}\left(\frac{A}{d}\right)\;\;$$

where *ε_0_* is the vacuum dielectric constant, *ε_r_* is the relative permittivity of the dielectric layer, and the value of *ε_r_* can be determined by the content of the filler material and the deformation of the dielectric layer under pressure. *A* is the area of the effective region and *d* is the distance between the upper electrode plate and the lower electrode plate. According to Equation (3), the incorporation of AgNWs can change the value of *ε_r_* of PDMS sponge dielectrics, and the change in *ε_r_* will be maximized when the load amount of AgNWs reaches a certain content. When external pressure acts on the sensor, the distance between the upper and lower electrodes of the sensor decreases, as does the distance between the silver nanowires adsorbed inside the PAP sponges. Therefore, the dielectric constant increases and the sensor capacitance increases. Because the PAP sponge composite dielectric layer is a porous material, the area, A, also increases. Due its low elastic modulus, the PAP sponge can undergo large deformation under low pressure, which increases the capacitance of the sensor. Simultaneously, when the PAP sponge is compressed by force, the distance between the silver nanowires adsorbed inside the PAP sponge decreases, and the dielectric constant increases, thereby increasing the capacitance of the sensor. These two effects combined give the PAP-sponge flexible capacitive sensor high sensitivity. In addition, the PAP sponge has excellent elasticity and strong recovery ability. After the external pressure is removed, its recovery speed is fast, so the response time of the sensor is short, and the repeatability is high.

**Figure 4:**
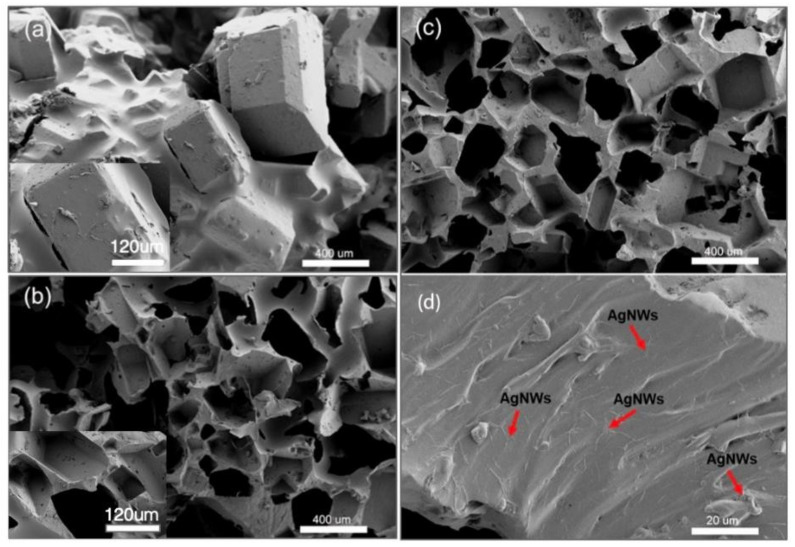
(**a**) SEM images of PDMS + sucrose. (**b**) SEM images of PDMS sponges. (**c**) SEM images of PAP sponges. (**d**) Final structure of porous PAP sponges. Insets are the high magnification SEM images.

SEM was performed on samples of the PAP sponge at different stages of its production to further characterize the microscale structural properties of the PAP sponge. Figure 4a shows the initial sucrose particles wrapped and bonded to PDMS. According to the enlarged part of Figure 4a, it can be clearly seen that PDMS adhered to the surface of the template, with the spaces between the particles filled with PDMS. Figure 4b shows that, as the sucrose template dissolved, the PDMS sponge developed a three-dimensional porous structure with relatively smooth pore walls, interconnected open pore structures and relatively independent closed pore structures. This was because the sucrose template had both complete bulk particles and relatively small sucrose clastic. When the template dissolved, two kinds of pore structures above were formed. As shown in Figure 4c, the loading AgNWs caused the pores of the sponge to become rougher. Figure 4d shows the final structure with AgNWs loaded onto the three-dimensional porous PAP sponge.

**Figure 5:**
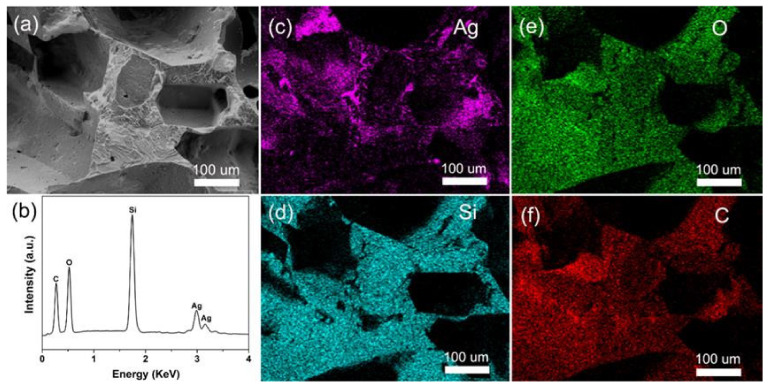
(**a**) SEM image of the P-3 PAP sponge. (**b**) Energy dispersive spectroscopy of the PAP sponge. Element maps were generated of silver (**c**), silicon (**d**), oxygen (**e**), and carbon (**f**).

Further analysis via energy dispersive spectroscopy was performed to explore the element distribution of P-3 sample. Elemental mapping of a section of PAP (Figure 5a) was performed to show the distribution of the individual elements throughout the pore structure. An energy spectrum of the PAP sponge (Figure 5b) showed that the only elements present at detectable levels were Si, O, C and Ag which demonstrated that AgNWs were loaded on the sponge skeleton. At the same time, it also showed that the surface layer of the PAP sponge was composed of Si, O, C and Ag.

**Figure 6:**
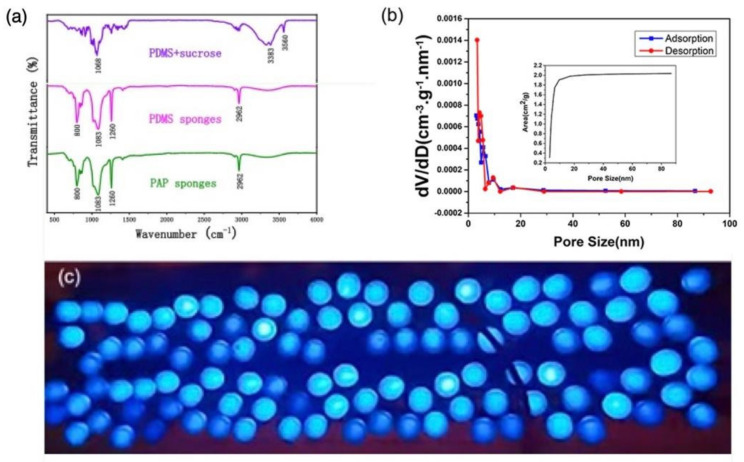
(**a**) Infrared spectra of PDMS + sucrose, PDMS sponges and PAP sponges; (**b**) the specific surface and porosity of PAP sponges (the illustration shows the variation in specific surface area of the PAP sponge with pore size). (**c**) 110 LED light bulbs lit up when pressed into the optimized PAP sponge capacitive pressure sensor.

Infrared analysis was conducted to further explore the characteristic peaks between the PAP sponge components at different stages of synthesis (Figure 6a). The 1068 cm^−1^, 3383 cm^−1^, and 3560 cm^−1^ peaks in the initial PDMS sponge with sucrose template corresponded to the stretching vibration of sucrose C-O and the O-H stretching vibration of hydroxyl, respectively. These characteristic peaks of sucrose disappeared after the template was dissolved and removed. Peaks at 800 cm^−1^, 1260 cm^−1^ and 2962 cm^−1^, corresponding to the stretching vibration of Si-CH_3_ bonds and a peak at 1083 cm^−1^, attributed to the adsorption of Si-O-Si bonds, were visible before and after AgNWs loading. There was no obvious change in the infrared absorption peak of the PDMS component of the samples before and after loading, indicating that PDMS and AgNWs were physically adsorbed rather non-chemically bonded.

The specific surface area of the P-3 sample was measured by BET (Brunner − Emmet − Teller) method with nitrogen as the adsorbate and helium as the carrier gas. The BET curve of the P-3 sample (Figure 6b) indicated that the diameters of AgNW-loaded pores in the PAP sponge were mostly in the range of 0–10 nm, and the average pore diameter was 5.546 nm, with a pore volume of 0.004 cm^3^/g. In addition, the illustration shows that the specific surface area improved with increase in the pore size in the range of 0–10 nm, up to 2.0 cm^2^/g. When the pore size was larger than 10 nm, the specific surface area tended to adopt a steady state at about 2.0 cm^2^/g.

Finally, to visually demonstrate the properties of the PAP sponge using a controllable test system, about 110 LED lights were shown to light up by pressing the P-3 capacitive pressure sensor (Figure 6c). The LEDs were clearly visible at night, highlighting the potential application of these polymer-material-based capacitive pressure sensors in flexible and wearable devices.

## 4. Conclusions

A porous PAP sponge was prepared by loading one-dimensional AgNW conductive filler onto PDMS sponge synthesized using a sugar template and coating the result with PDMS film. Through this simple and cost-effective process, we were able to fabricate flexible and ultrasensitive capacitive pressure sensors. Varying this process to optimize capacitive response, we found that a PAP sponge made using 100% sucrose as the template with a 150 mg AgNWs loading capacity produced the best results (0.62 kPa^−1^). Characterization of the optimized PAP sponge revealed that the specific surface area of PAP sponge was able to reach 2.0 cm^2^/g in the range of 0–10 nm pore size, and that its tensile strength was as high as 1.425 MPa with elongation of 156.38%. In summary, the sponge exhibited excellent waterproof performance, high elasticity, low hysteresis, and low weight, and overall low density. As demonstrated by its ability to light up 110 LED lights under slight pressure, the PAP sponge described here has many potential applications in the fields of flexible electronics and wearable devices.

## Figures and Tables

**Table 1:** A comparison of performance between our study and other types of pressure sensors previously reported.

Reference	Sensitivity [kPa^−1^]
[This work]	0.62
[67]	0.58
[68]	0.55
[65]	0.26
[69]	0.21
[66]	0.11
[70]	0.05

## Data Availability

The datasets of this study are available from the author on reasonable request.
